# Maxillary dentoalveolar assessment following retraction of maxillary incisors: a preliminary study

**DOI:** 10.1590/2177-6709.21.5.082-089.oar

**Published:** 2016

**Authors:** Tiago Maia Fernandes Oliveira, Lígia Vieira Claudino, Cláudia Trindade Mattos, Eduardo Franzotti Sant'Anna

**Affiliations:** 1 MSc student, Universidade Federal do Rio de Janeiro (UFRJ), Department of Pediatric Dentistry and Orthodontics, Rio de Janeiro, Rio de Janeiro, Brazil.; 2 PhD resident, Universidade Federal do Rio de Janeiro (UFRJ), Department of Pediatric Dentistry and Orthodontics, Rio de Janeiro, Rio de Janeiro, Brazil.; 3 Professor, Universidade Federal Fluminense (UFF), Department of Dental Clinics, Niterói, Rio de Janeiro, Brazil.; 4 Associate Professor and Chairman, Universidade Federal do Rio de Janeiro (UFRJ), Department of Pediatric Dentistry and Orthodontics, Rio de Janeiro, Rio de Janeiro, Brazil.

**Keywords:** Alveolar bone loss, Root resorption, Tooth movement, Cone-beam computed tomography

## Abstract

**Objective:**

The aim of this preliminary study was to assess changes in tooth length and alveolar thickness following retraction of maxillary incisors.

**Methods:**

A total of 11 patients presenting severe maxillary dentoalveolar protrusion revealed by initial (T_1_) cone-beam computed tomography (CBCT), and whose treatment plan included extraction of maxillary first premolars and retraction of maxillary incisors, were selected and submitted to CBCT examination one month after the end of incisors retraction (T_2_). The premaxilla was assessed through seven axial slices by means of Dolphin Imaging^TM^ software. In each of these slices, five measurements of the distance from the buccal cortical bone to the palatal cortical bone were performed. Tooth length of maxillary incisors (n = 44) was also measured in sagittal slices. Measurements were repeated after a two-week interval, and intraclass correlation coefficient (ICC) was used to test examiner calibration. Wilcoxon test was used to detect differences in measurements performed at the two time intervals.

**Results:**

The ICC was satisfactory for tooth length (0.890) and for premaxilla alveolar thickness measurements (0.980). Analysis of data showed no statistically significant differences (*p* > 0.05) in tooth length or alveolar thickness between the two-time intervals assessed.

**Conclusion:**

The force used in retraction of maxillary incisors in this research did not promote significant changes in tooth length of maxillary incisors or in premaxilla alveolar thickness.

## RESUMO

**Objetivo:**

o objetivo deste estudo preliminar foi avaliar as alterações no comprimento dentário e na espessura alveolar durante a retração dos incisivos superiores.

**Métodos:**

um total de 11 pacientes apresentando protrusão dentoalveolar severa, de acordo com a tomografia computadorizada de feixe cônico (TCFC) inicial (T_1_), e que continham no seu plano de tratamento extração dos pré-molares superiores e retração dos incisivos superiores, foram selecionados e submetidos a exames de TCFC um mês após o término da retração dos incisivos (T_2_). Com o auxílio do *software* Dolphin Imaging^(r)^, a prémaxila foi dividida em sete cortes axiais. Em cada corte axial, foram realizadas cinco medições da distância da cortical vestibular a lingual. O comprimento dos incisivos superiores (n = 44) foi medido no corte sagital. As medições foram refeitas após o intervalo de duas semanas e o coeficiente de correlação intraclasse (ICC) foi utilizado para avaliar a calibração do examinador. Utilizou-se o teste de Wilcoxon para detectar diferenças nas medições realizadas nos dois intervalos de tempo.

**Resultados:**

o ICC mostrou-se satisfatório tanto para o comprimento dentário (0,890) quanto para a espessura alveolar da pré-maxila (0,980). Não foram observadas diferenças estatísticas significativas para comprimento dentário e para as espessuras alveolares (*p* < 0,05) nos períodos avaliados.

**Conclusões:**

as forças atuantes na retração dos incisivos, nessa pesquisa, não provocaram alterações significativas no comprimento dentário dos incisivos superiores, nem mudanças na espessura alveolar da pré-maxila.

## INTRODUCTION

The current objectives of orthodontic treatment are based, among other factors, in the quest for adequate occlusion and esthetics associated with long-term maintenance of results.^1^ In specific cases, extensive movement of incisors is necessary to accomplish these goals.

In this context, several factors of mechanical and biological nature must be considered. From a biological point of view, topography of the alveolar bone, presence of dehiscence or fenestration, root length, tooth position, soft tissues condition and other aspects should be observed to avoid undesirable damage caused by moving teeth beyond the anatomic boundaries.^2^

An example of an undesirable effect of extensive retraction of maxillary incisors is the increase in thickness of the buccal cortical bone, which may result from lack of balance between bone resorption and neoformation, and depends on the amplitude, direction and quantity of movement, as well as on changes in tooth tipping.^3^

Cephalometric radiograph is a resource widely used by orthodontists as an auxiliary tool in orthodontic diagnosis and treatment plan. However, it presents as its main limitations a considerable amount of distortion, superimposition of structures, and difficulty identifying changes in the midface.^4^ Nevertheless, due to the limitations described, studies considering the tridimensional aspect of the dentoalvolar structure are necessary.

With the advent of cone-beam computed tomography (CBCT), achieving images of craniofacial structures with good accuracy has become possible. These images are used as an aid in treatment plan of patients in need of complex orthodontic treatment, as they allow assessment of tridimensional morphological changes resulting from treatment and/or growth.^5^ Additionally, they allow distinction and measurement of tooth root proximity with cortical bone and follow-up of root resorption.^6^

Previous studies have validated CBCT for quantitative analysis of important aspects related to the dentoalveolar complex, showing high accuracy and precision of measurements.^7^ ^,^ ^8^ This accuracy is associated with image clearness and resolution.^9^ Spatial resolution obtained by CBCT depends, among other factors, on voxel dimension, which represents the smallest image unit. The lower the dimension of the voxel, the greater the resolution of the image and the greater the radiation dose, which is a disadvantage.^10^ ^,^ ^11^

Therefore, the objective of this study was to assess potential changes in dentoalveolar structures, following retraction of maxillary incisors, in patient submitted to first premolars extraction through CBCT.

## MATERIAL AND METHODS

This prospective research was approved by the Ethics Committee of Universidade Federal do Rio de Janeiro (UFRJ) Institute of Studies in Collective Health. All subjects included in the study read and signed an informed consent form.

Sample size calculation was performed based on the maximum standard deviation set in a previous study,^12^ considering a test power of 0.80 and α = 0.05. Calculation showed that ten patients would be necessary to detect a difference of 2.5 mm of incisors root resorption. The formula used was described by Pandis.^13^

A total of 11 patients subjected to treatment with Edgewise standard fixed appliances in the graduate orthodontic clinics of Universidade Federal do Rio de Janeiro (UFRJ), School of Dentistry, were selected and included in this research. Eight patients presented with Class I malocclusion and three presented Class II, Division 1 malocclusion. Six patients were women and five were men, with patients' age ranging from 18 to 26 years old. Patients were selected after having their clinical records examined and cephalometric data obtained from initial CBCT examination. Inclusion criteria were: 1-NA distance higher than 4 mm; 1-NA angle higher than 22^o^; interincisal angle lower than 130^o^; clinical evidence of dentoalveolar protrusion in the maxilla; maxillary crowding; no history of trauma and incisors root resorption before treatment; treatment plan including extraction of maxillary first premolars and complete retraction of maxillary canines and incisors to correct protrusion and inclination. Exclusion criteria were: diseases, pathologies or drug treatment that could affect bone metabolism.

CBCT is an examination requested as part of the initial records for orthodontic diagnosis and treatment plan in the graduate orthodontic clinics of Universidade Federal do Rio de Janeiro (UFRJ), School of Dentistry. Therefore, patients selected to take part in the research already presented clinical records and initial CBCT examination (T_1_). 

Patients were submitted to the same orthodontic treatment protocol: from the setup of standard Edgewise metal brackets to the complete retraction of maxillary incisors which was carried out by means of extraction of maxillary first premolars, alignment and leveling phase, as well as retraction of canines with elastomeric chain. The archwire used for incisors retraction was a 0.019 x 0.025-instainless steel wire with omega loops distal to the brackets of first molars, and 6-mm long teardrop loops distal to lateral incisors. Activation of incisors retraction archwire was performed after each 21-day interval with stainless steel ligature tie-back on the omega loops to promote a 0.5 to 1-mm opening of the loops, generating a force of 150 gf per side. Tipping and extrusion of maxillary incisors during retraction were controlled by incorporating active torque on incisors (gable effect). Anchorage was controlled by a transpalatal arch and/or extraoral traction when maximum anchorage was required. The mean retraction time was six months. The amount of retraction was similar for all patients, once the orthodontic mechanics used was standardized. One month after the end of complete retraction of maxillary incisors, an additional CBCT (T_2_) was requested.

Tomographic examination was performed by means of i-CAT scan (Imaging Sciences International, Hatfield, Pennsylvania, USA) set at 120 kVp, 5 mAs, 13 x 17 cm FOV, 0.4 mm voxel, and scanning time of 20 seconds. Data obtained were recorded in DICOM (Digital Imaging and Communication in Medicine & Management Solutions) format and assessed by means of Dolphin Imaging^TM^ software version 11.5 (Dolphin Imaging, Chatsworth, California, USA). 

**Figure 1:**
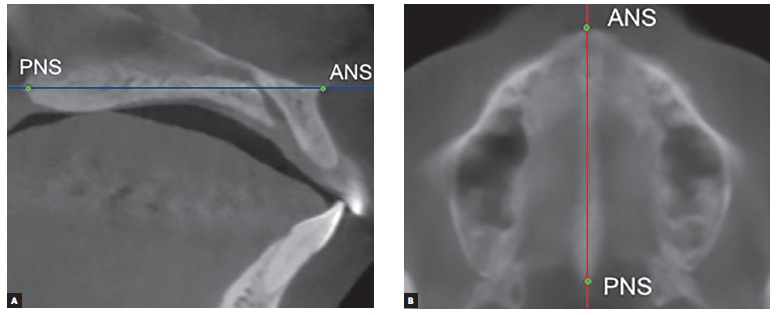
A) Sagittal slice showing ANS and PNS (green points) coinciding with the axial plane (blue line). B) Axial slice showing ANS and PNS (green points) coinciding with the sagittal plane (red line).

**Figure 2:**
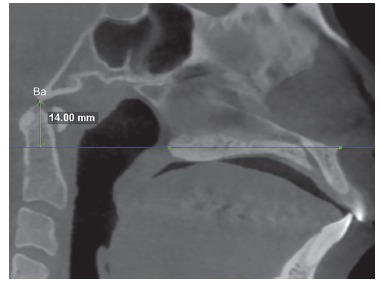
Distance from basion to the palatal plane (blue line) extended posteriorly.

**Figure 3:**
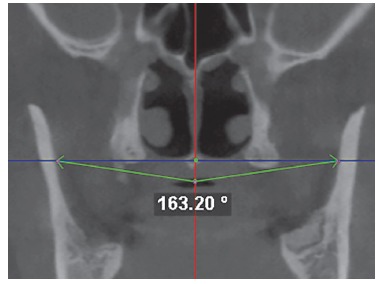
Angle between two points determined in the internal face of the mandibular right and left ramus with the third point 5 mm below the PNS parallel to the sagittal plane (red line).

Volumetric reconstruction was obtained for each tomographic scan with standardized head position.^14^ Additionally, head orientation was more specifically confirmed and adjusted through marking anatomical landmarks in multiplanar images and measuring specific distances and angles. This procedure allowed reproducibility of measurements in the two time intervals for each patient. For that purpose, in the midsagittal slice, the anterior nasal spine (ANS) and the posterior nasal spine (PNS) were marked, and the palatal plane rotated, if necessary, until it was parallel to the axial plane. The axial slice correspondent to the palatal plane was located and the image rotated, if necessary, until the line uniting ANS and PNS was parallel to the sagittal plane (Fig 1). In the sagittal slice where ANS and PNS were visible, the distance from the basion to the palatal plane extending posteriorly was measured (Fig 2). This distance should be the same in the two CBCT scans obtained from the same patient (T_1_ and T_2_). In the coronal slice where the PNS was visible, two reference points were determined in the internal face of the mandibular right and left ramus in the same axial plane of PNS. A new reference point was marked 5 mm below the PNS parallel to the sagittal plane. The angle between these three points had to be the same in the two CBCT scans obtained from the same patient (T_1_ and T_2_) (Fig 3).

**Figure 4:**
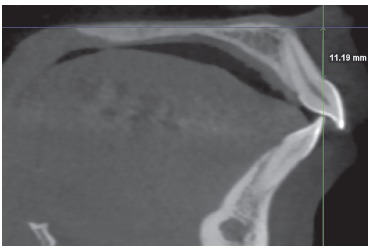
Distance from the cementoenamel junction (CEJ) to the palatal plane.

Assessment of alveolar structures was performed in seven axial slices parallel to the palatal plane. In the initial CBCT, in the sagittal slice that passes through the middle of the crown of the maxillary right central incisor, the distance from the cementoenamel junction (CEJ) to the palatal plane was measured (Fig 4). The first axial slice selected was 2 mm above the CEJ and the six subsequent slices were above the first one, 2 mm apart from each other. The distance measured in the initial CBCT was reproduced in the post-retraction CBCT of the same patient with its start in the palatal plane and its end near or on the CEJ (depending on the vertical movement of the tooth during retraction). The first axial slice was selected 2 mm above this point, so that the same alveolar structures could be compared.

**Figure 5:**
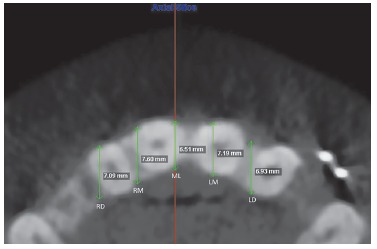
Measures of bone thickness in five distinct regions used in the axial slices: the first one in the midsagittal plane (ML-midline), and the other ones 5 and 10 mm apart from ML to the right (RM and RD) and to the left (LM and LD).

In the axial slices, thickness of the alveolar process between the buccal and palatal cortical bone was measured in five distinct regions: the first one in the midsagittal plane (ML), and the others 5 and 10 mm apart from ML to the right (RM and RD) and to the left (LM and LD) (Fig 5). A total of 35 measurements of alveolar thickness were computed for each CBCT.

**Figure 6:**
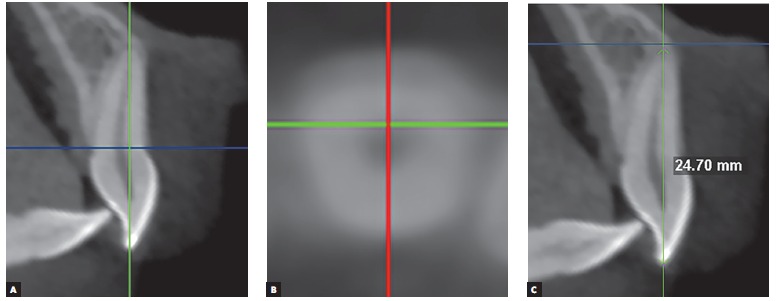
A) Sagittal slice. Coronal plane passing through the long axis of the incisor to be measured (green line), axial plane (blue line) passing through the cervical portion of the root; B) Axial slice. Sagittal plane (red line) passing through the middle of the root canal in the buccolingual direction; C) Sagittal slice. Distance between the root apex and the incisal border

For tooth length assessment (n = 44), the sagittal slice selected was the one passing through the long axis of the incisor to be measured. In the sagittal slice, the image was rotated until a plane parallel to the coronal plane passed through the root apex and the most buccal point in the incisal border. An axial slice passing through the cervical portion of the root was selected (Fig 6A). In the axial slice, the image was rotated until the sagittal plane passed through the middle of the root canal in buccolingual direction (Fig 6B). In the correspondent sagittal slice, the distance between the root apex and the incisal border was measured (Fig 6C).

### Statistical analysis

Intraclass correlation coefficient (ICC) tests were performed to assess examiner calibration. 

Shapiro-Wilk test was performed to check for normality of data. Since the hypothesis of normality was rejected, a nonparametric Wilcoxon test was used to compare pre- and postretraction measurements. A level of significance of 0.05 was adopted.

## RESULTS

The inicial mean of 1-NA distance, 1-NA angle and interincisal angle were 9.2 mm, 31.5° and 104.6°, respectively. 

ICC was 0.899 for tooth length and 0.980 for alveolar thickness measurements, thus confirming examiner calibration. 

Table 1 presents the descriptive statistics for tooth length measurement of the four maxillary incisors. The length of incisors tended to present a very small decrease, but differences were not statistically significant.

**Table 1:** Descriptive analysis of tooth length (mm) and *p*-value of Wilcoxon test.

Tooth	Period	Mean (SD)	Minimum	Maximum	*p*-value
Right lateral incisor	T_1_	23.06 (2.37)	19.64	25.83	0.310
	T_2_	22.22 (2.21)	19.08	25.04	
Right central incisor	T_1_	25.01 (2.04)	22.11	27.43	0.220
	T_2_	24.21 (2.33)	21.46	27.06	
Left central incisor	T_1_	25.04 (1.95)	22.68	27.65	0.370
	T_2_	24.24 (2.28)	21.34	27.58	
Left lateral incisor	T_1_	22.98 (2.06)	19.09	25.47	0.250
	T_2_	22.09 (2.45)	18.03	24.86	

Table 2 presents the descriptive statistics for alveolar thickness measurements. Some small degree of variation could be observed, and alveolar thickness either increased or decreased. However, differences were not statistically significant.

**Table 2:**
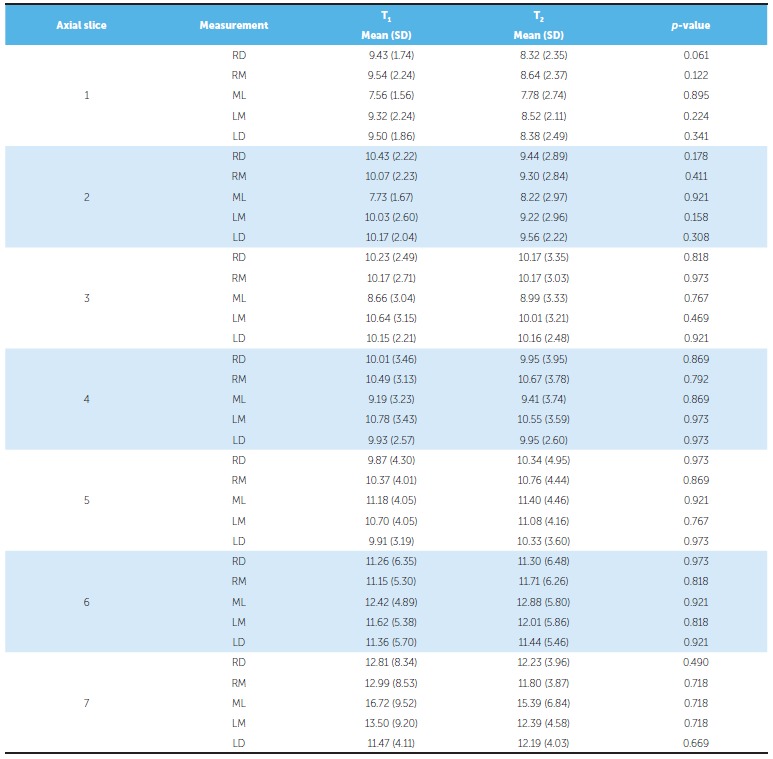
Descriptive analysis of alveolar bone thickness of the maxilla (mm) and *p*-value of Wilcoxon test.

Measures of alveolar thickness between as vestibular and lingual corticals: ML = measure at median sagittal plane; RM and RD = measures at 5 and 10mm, respectively, to the right side; LM and LD= measures at 5 and 10mm, respectively, to the left side.

## DISCUSSION

Our results show a mean decrease of less than 1 mm in tooth length of the four maxillary incisors. These changes were not statistically significant. These findings differ from other results reported in the literature by authors who studied root resorption associated with orthodontic treatment and reported greater mean root resorption.^12^ ^,^ ^15^ However, patients in our study were assessed immediately after the end of incisor retraction and not after the end of orthodontic treatment, which may have contributed to the results observed. Root resorption during orthodontic treatment may be associated with factors such as individual predisposition, magnitude and direction of tooth movement, root anatomy and shape, need for premolar extraction, presence of root resorption previous to treatment, and treatment time.^15^ ^,^ ^16^ ^,^ ^17^

One of the advantages of CBCT in Orthodontics is to assess and measure buccal and lingual bones surrounding the teeth with lower radiation dose than CT scans, considering that these structures could not be assessed by conventional radiograph due to imaging overlap and gingival covering.^18^

In CBCT examination used in Orthodontics, greater FOVs are generally used, which makes it impossible to work with voxels smaller than 0.3 mm due to greater radiation doses.^19^ In this research, greater voxel size was used to minimize radiation dose and follow the ALARA principle (As Low As Reasonably Achievable). Some studies show that smaller voxels may lead to better accuracy in measurements;^9^ ^,^ ^11^ however, there are authors who confirm the efficacy of using CBCT scans to measure small or delicate bone thickness and bone or periodontal defects with 0.38 to 0.4 mm of voxel size.^6^ ^,^ ^20^ ^,^ ^21^ In our study, 0.4-mm-voxel images were used. One of the difficulties in measuring bone structures with CBCT scans is image clearness. According to Molen et al,^10^ the thinner the bone plate is, the less distinct the image is, which decreases precision of linear measurements. This limitation may be due to the partial volume averaging property, which happens when the limit between two tissues of different density lies in a voxel. Density in this specific voxel will correspond to the mean between the densities of the two structures. When the sum of many measures may be affected by this property, this may produce significant differences from the actual measures.^22^ Another phenomenon known to cause alteration in measures is the limitation of contrast resolution described by Ballrick et al.^23^ This limitation is due to the incapacity of distinguishing two objects of similar density when they are too close. The authors concluded that a minimum distance of 0.86 mm was necessary to assure clear distinction between two metal plates of the same density. All patients included in this research had metal brackets bonded to teeth and evaluation was not hindered by any imaging artifacts. 

In this research, assessment of alveolar changes in the maxilla was performed through linear measurements between buccal and palatal cortical bones. These measures are independent and are considered long enough not to be influenced by variation in the partial volume averaging. Additionally, voxel size was the same for all tomographic exams used in this study. That means that if there has been a tendency towards overestimating measures, this has happened to all measures, so that the differences between pre- and postretraction values would have been the same and the results would not have been altered. The limitation of contrast resolution would probably have introduced some bias, if bone thickness had been measured from the buccal and palatal surfaces of incisors roots in their cervical third, due to the smaller quantity of alveolar bone in that region.

A few studies in the literature present changes in alveolar bone thickness following incisors retraction, using computed tomography. As our objective was to assess changes in alveolar bone thickness from buccal to palatal cortical bone, this distance was measured in a standardized and reproducible manner. We did not measure the distances from the tooth root to the buccal cortical bone and from the tooth root to the palatal cortical bone as other authors did,^2^ ^,^ ^24^ since this measure reflects mainly the displacement of the tooth root through the alveolar bone and not specifically changes in bone thickness of the region of interest. Our study presented small non-significant differences in alveolar bone thickness, which either decreased or increased. The greatest difference was 1.33 mm. These results may indicate that no undesirable thickening occurred in the cortical bone, which could hinder esthetic results achieved as a result of treatment. The findings by Sarikaya et al^2^ are not comparable to ours, as their measurements were different, but they report bone loss after retraction, especially in the palatal alveolar bone. Yodthong, Charoemratrote and Leethanakul^3^ assert that changes in alveolar bone thickness during orthodontic treatment with retraction of maxillary incisors may be related to the amount of tooth movement, changes in tooth tipping and further intrusion of incisors.

However, when incisors are retracted, the risk of alveolar bone loss should be considered, and therapeutic limitations of orthodontic tooth movement should be greatly emphasized.^3^ In our study, changes in alveolar thickness measurements were not statistically significant, and these results may be related to controlled force as well as to the mechanics used.

This is a preliminary study of which findings should be considered with caution. Statistical differences between dentoalveolar structures before and after retraction of maxillary incisors could have been observed if sample size was greater. The need for additional studies, especially RCTs assessing dentoalveolar changes associated with orthodontic treatment with CBCT scans using reduced voxels and limited FOV, is evident.

## CONCLUSION

Based on the results from this preliminary study, we conclude that there were no significant changes in tooth length of the four maxillary incisors and in alveolar bone thickness after retraction of maxillary anterior teeth.
